# Role of Germination in Murine Airway CD8^+^ T-Cell Responses to *Aspergillus* Conidia

**DOI:** 10.1371/journal.pone.0018777

**Published:** 2011-04-13

**Authors:** Steven P. Templeton, Amanda D. Buskirk, Brandon Law, Brett J. Green, Donald H. Beezhold

**Affiliations:** 1 Allergy and Clinical Immunology Branch, Health Effects Laboratory Division, National Institute for Occupational Safety and Health, Centers for Disease Control and Prevention, Morgantown, West Virginia, United States of America; 2 Department of Microbiology, Immunology, and Cell Biology, West Virginia University School of Medicine, Morgantown, West Virginia, United States of America; University of Minnesota, United States of America

## Abstract

Pulmonary exposure to *Aspergillus fumigatus* has been associated with morbidity and mortality, particularly in immunocompromised individuals. *A. fumigatus* conidia produce β-glucan, proteases, and other immunostimulatory factors upon germination. Murine models have shown that the ability *of A. fumigatus* to germinate at physiological temperature may be an important factor that facilitates invasive disease. We observed a significant increase in IFN-γ-producing CD8^+^ T cells in bronchoalveolar lavage fluid (BALF) of immunocompetent mice that repeatedly aspirated *A. fumigatus* conidia in contrast to mice challenged with *A. versicolor*, a species that is not typically associated with invasive, disseminated disease. Analysis of tissue sections indicated the presence of germinating spores in the lungs of mice challenged with *A. fumigatus*, but not *A. versicolor*. Airway IFN-γ^+^CD8^+^ T-cells were decreased and lung germination was eliminated in mice that aspirated *A. fumigatus* conidia that were formaldehyde-fixed or heat-inactivated. Furthermore, *A. fumigatus* particles exhibited greater persistence in the lungs of recipient mice when compared to non-viable *A. fumigatus* or *A. versicolor*, and this correlated with increased maintenance of airway memory-phenotype CD8^+^ T cells. Therefore, murine airway CD8^+^ T cell-responses to aspiration of *Aspergillus* conidia may be mediated in part by the ability of conidia to germinate in the host lung tissue. These results provide further evidence of induction of immune responses to fungi based on their ability to invade host tissue.

## Introduction

Filamentous fungi are ubiquitous microorganisms in indoor and outdoor environments and acquire nutrients from a wide variety of substrates such as decaying plant matter or water-damaged building materials [1]. The small size of conidia (i.e. asexual spores) of many fungal species allows particles to easily become airborne and inhaled, with a potential for deposition in the terminal airways of the lungs. Small amounts of inhaled conidia are quickly phagocytosed and degraded by alveolar macrophages [2], [3]. However, repeated exposure to large numbers may result in persistence of conidia and induction of airway inflammation.

Conidia from the genus *Aspergillus* have been associated with allergic sensitization as well as exacerbation of allergy and asthma in otherwise healthy individuals [2], [3]. However, the pathology of *Aspergillus*-associated invasive disease varies between fungal species. *Aspergillus fumigatus* is the etiologic agent of allergic bronchopulmonary aspergillosis (ABPA), has been associated with hypersensitivity pneumonitis, and is a primary cause of invasive aspergillosis in immunocompromised individuals [3]. In contrast, *A. versicolor* is not typically associated with invasive pulmonary infection. The ability of *A. fumigatus* to colonize the respiratory tract of susceptible individuals has been attributed to several biological properties. In contrast to *A. versicolor*, *A. fumigatus* can maintain growth within a wide range of temperatures, from below 20°C up to 70°C [3], [4]. Furthermore, *A. fumigatus* conidia exhibit an ability to persist inside macrophages after phagocytosis or produce factors that inhibit phagocytosis [5], [6], [7]. Conidia that persist in the lungs of immunocompromised individuals may germinate and form hyphal structures that invade surrounding tissue. Furthermore, release of immunostimulatory molecules such as β-glucan and allergens have been shown in germinating, but not resting, conidia [8], [9]. The ability of *A. fumigatus* to exhibit invasive growth in the respiratory tract is believed to be mediated in part by the ability to germinate at physiological temperatures and by the secretion of fungal proteases [7]. In support of these hypotheses, recent studies of gene-targeted mutants have demonstrated that decreased thermotolerance or protease secretion resulted in significantly decreased virulence of *A. fumigatus* in murine models of invasive infection [10], [11]. Based on the results of these studies, lung persistence and tissue invasion are characteristics of *Aspergillus* conidia that may be species-dependent.

Both innate and adaptive immunity are critical in the development of immune protection from invasive aspergillosis. In addition to phagocytosis by resident alveolar macrophages, inhaled *A. fumigatus* conidia are prevented from germination and the establishment of early invasive infection by infiltrating neutrophils [12], [13]. However, adaptive immune responses also provide protection from invasive infection. Adoptive transfer of fungal-specific CD4^+^ T_h_1 lymphocytes confers protection from infection in mice [14], and in humans [15]. *A. fumigatus*-specific CD4^+^ [16], [17], [18] and CD8^+^ T cells [17] have also been isolated from healthy patients, suggesting that routine exposure to *A. fumigatus* may confer protective immunity. Although CD4^+^ T cells are considered the primary effector cell in protective immunity, the role of CD8^+^ T cells in protection from respiratory infection with *A. fumigatus* remains unknown.

In *A. fumigatus*-challenged mice, fungal-specific, IFN-γ-producing CD4^+^ T cells were recruited to the airways of mice that received a single intratracheal instillation of a large dose (20×10^8^) of viable spores [19]. Another recent study demonstrated increased lung tissue T_h_1, T_h_2, and T_h_17 responses and infiltration of CD8^+^ T cells with repeated intranasal challenges of smaller doses (2×10^6^) *A. fumigatus* conidia [20]. However, since the ability to persist and germinate in the host may vary between *Aspergillus* species, it is possible that the airway immune responses are also fungal species-dependent. In this study, we aimed to further examine the induction and maintenance of airway CD8^+^ T-cell responses to repeated exposures of *A. fumigatus* conidia. Airway T-cell responses to *A. fumigatus* were composed of IFN-γ-producing CD4^+^ and CD8^+^ T cells, whereas airway responses to *A. versicolor* were predominantly CD4^+^ T cell-mediated. Airway CD8^+^ T-cell responses to *A. fumigatus* were partly dependent on the ability of conidia to germinate, and this correlated with persistence of conidia in the lungs and maintenance of airway memory-phenotype CD8^+^ T cells. These results suggest that airway immune responses are programmed in response to fungal factors such as the ability to germinate in host lung tissue.

## Results

### Murine airway responses to repeated aspiration of *Aspergillus* conidia

**Figure 1:**
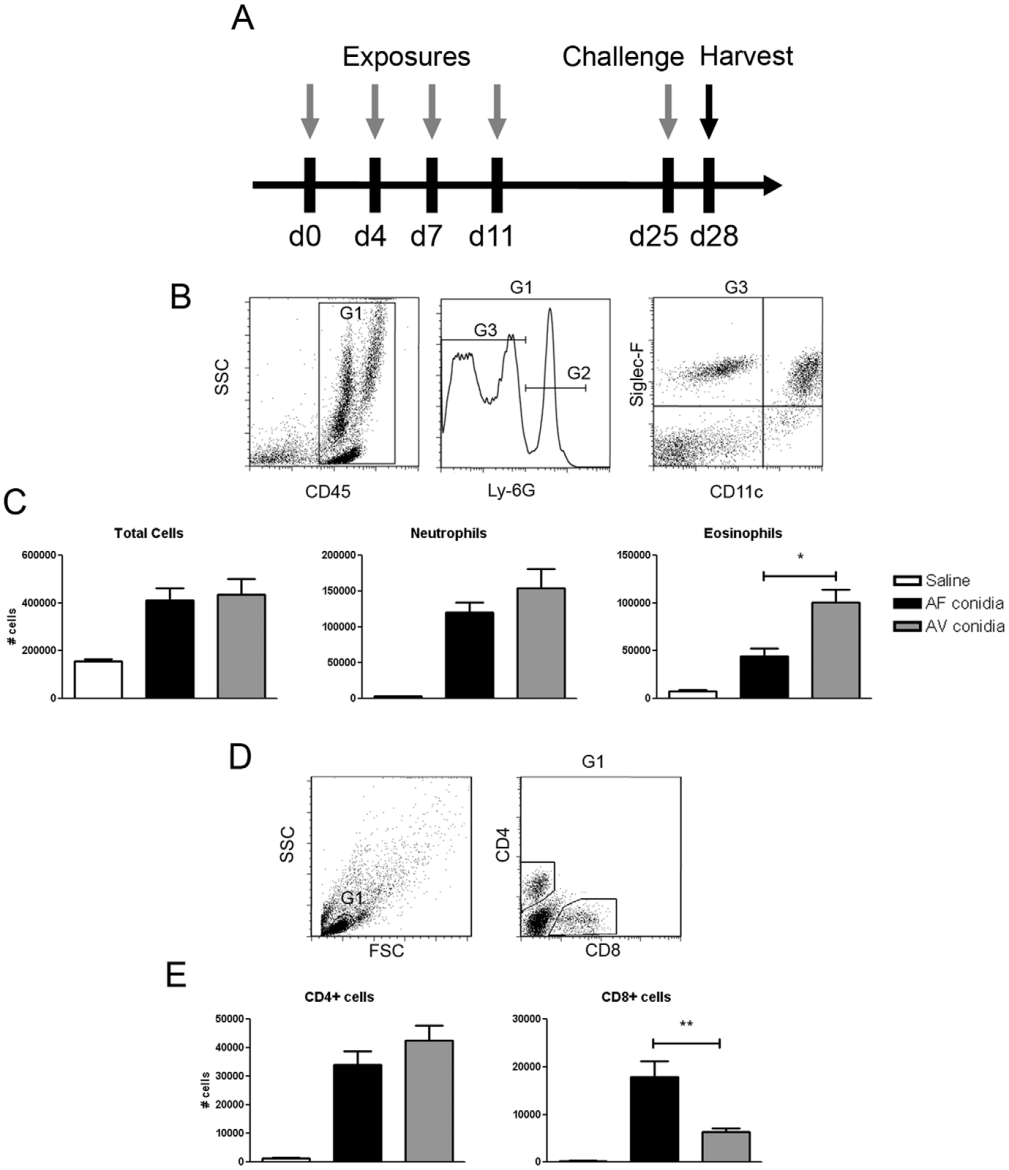
Airway leukocyte recruitment in mice exposed to repeated aspiration of *Aspergillus* conidia. 1A, BALB/c mice aspirated 2×10^6^ *Aspergillus* conidia in suspension at the time points indicated. B, Representative flow cytometric dot plots with granulocyte gating of BALF. Neutrophils were defined as CD45^hi^Ly-6G^hi^CD11c^lo^ (G2). Eosinophils were defined as Ly-6G^lo^Siglec-F^hi^CD11c^lo^ in order to distinguish these cells from Ly-6G^lo^Siglec-F^hi^CD11c^hi^ alveolar macrophages (G3). C, total # of BALF cells, neutrophils, and eosinophils. D, Representative flow cytometric gating of lymphocytes (left panel), with CD4^+^ and CD8^+^ populations (right panel). E, BALF T-cell composition after repeated aspiration of *A. fumigatus* or *A. versicolor* conidia. Data depicted in C, E, are a summary of two experiments with 6–11 mice per group. *p<0.01, **p<0.05.

Using two *Aspergillus* species (*A. fumigatus* and *A. versicolor*), we established a murine model of repeated pharyngeal aspiration (Figure 1A). Aspirated BALB/c mice were then rested for 2 weeks and sacrificed 3 days after a final challenge. BALF from each animal was analyzed by flow cytometry to measure airway leukocyte recruitment. The frequency of each population and total number of cells collected were used to calculate the total number of airway neutrophils, eosinophils, and CD4 and CD8 T cells (Figure 1B). Airway infiltration of CD45^hi^ leukocytes was increased in mice that aspirated conidia of *A. fumigatus* or *A. versicolor* (Figure 1C, left panel). Ly-6G^hi^ neutrophils were also increased in both exposure groups (Figure 1C, middle panel). Although aspiration of conidia increased airway eosinophils in both groups, mice that aspirated *A. versicolor* exhibited higher numbers in comparison to *A. fumigatus* aspirated mice (Figure 1C, right panel). Recruitment of CD4^+^ and CD8^+^ T lymphocytes were also analyzed by flow cytometry (Figure 1D, E). Although mice that aspirated conidia from either species of *Aspergillus* exhibited increased airway CD4^+^ T cells (Figure 1E, left panel), CD8^+^ T cells were more significantly increased in mice that aspirated *A. fumigatus*. This suggests that leukocyte populations recruited to the airways in response to aspiration of *Aspergillus* conidia varies between species.

**Figure 2:**
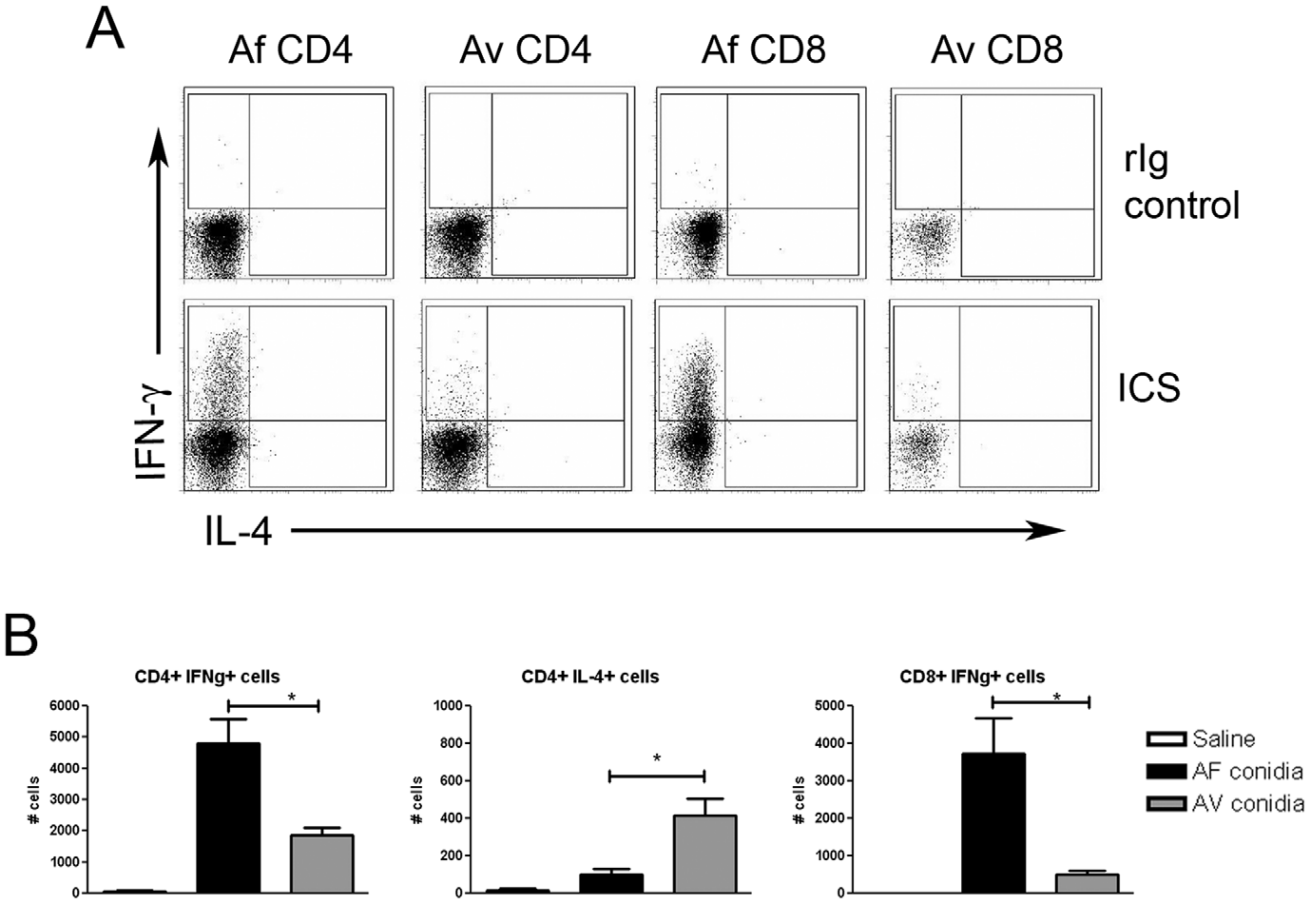
BALF T-cell cytokine production after repeated aspiration of *Aspergillus* conidia. A, flow cytometric dot plots of IFN-γ and IL-4 production in BALF CD4 and CD8 T cells. Representative dot plots are from parent gates similar to those depicted in Figure 1D. B, Total # of IFN-γ and IL-4-producing CD4^+^ T cells and IFN-γ-producing CD8^+^ BALF T cells. Data depicted in B are a summary of two experiments with 6–11 mice per group. *p<0.05.

To examine the effector functions of murine airway T cells in response to *Aspergillus* conidia, we performed *ex vivo* intracellular cytokine staining on BALF cells that had been stimulated with PMA/Ionomycin for 4 hours at 37°C in the presence of Brefeldin A. We examined intracellular production of IFN-γ and IL-4 in BALF CD4^+^ and CD8^+^ T cells (Figure 2A, bottom panels) in comparison to BALF cells stained with control rat-Ig (Figure 2A, top panels). Intracellular production of IFN-γ was significantly increased in both CD4^+^ and CD8^+^ T-cells in mice that aspirated *A. fumigatus* (Figure 2B, left and right panels, respectively). However, in response to *A. versicolor*, fewer CD4^+^ T cells produced IFN-γ. Low numbers of IL-4-producing CD4^+^ T cells were detected, although the number of IL-4-producing cells was increased in response to *A. versicolor* when compared to *A. fumigatus* (Figure 2B, middle panel). Therefore, in addition to differences in leukocyte recruitment, airway T-cell cytokine production in mice that aspirated *Aspergillus* conidia may vary between different species.

### Germination of inhaled *Aspergillus fumigatus* conidia in the lungs

**Figure 3:**
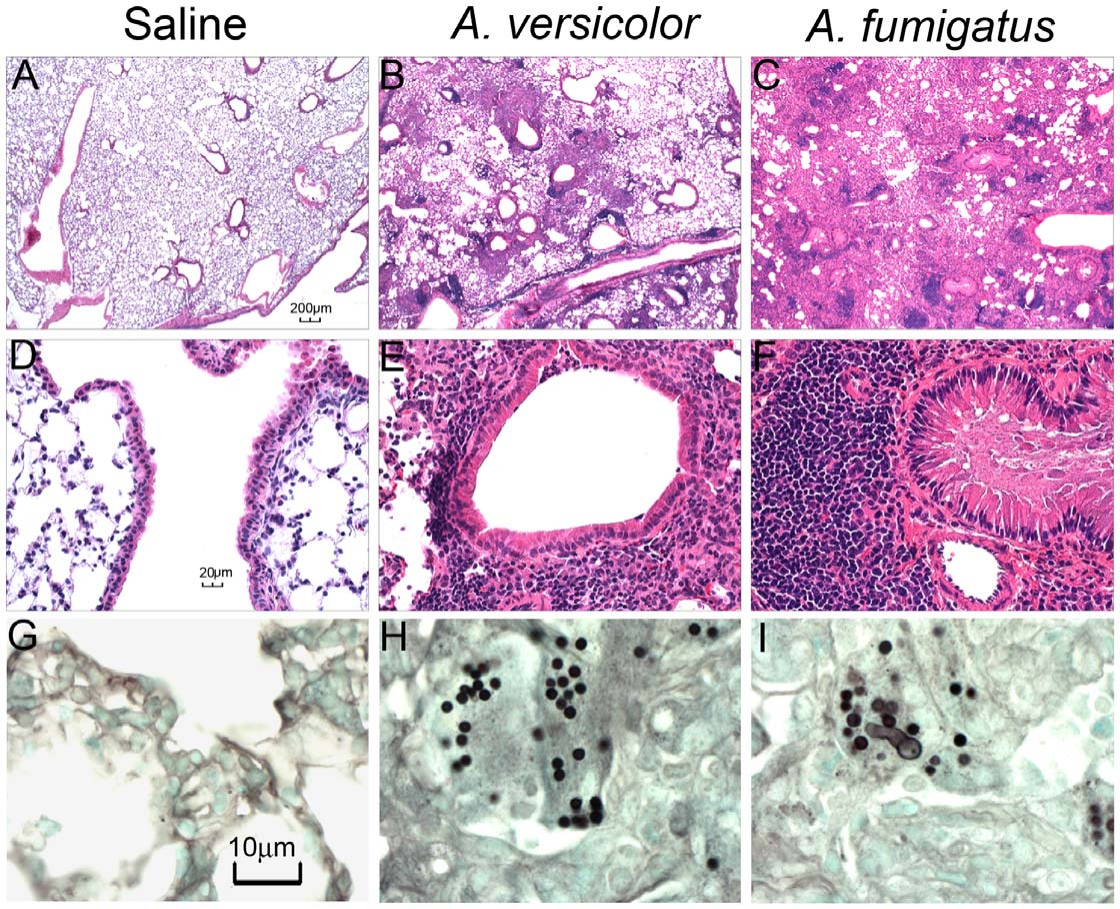
Lung histopathology and germination of conidia in mice exposed to *Aspergillus* conidia. A–F, H&E staining of mouse lung tissue. G–I, GMS staining of lung tissue. A,D,G, saline controls. B,E,H, *A. versicolor*, C,F,I, *A. fumigatus*. A–C 4× magnification, D–F, 40× magnification. G–I, 100× magnification. Data depicted are representative samples of 5 mice per group with one lung section analyzed per animal.

Next we compared the ability of *Aspergillus* conidia to germinate in the lungs. In comparison to control mice (Figure 3A, D), animals that aspirated *A. versicolor* (Figure 3B, E), or *A. fumigatus* (Figure 3C,F) exhibited airway inflammation and bronchiocentric infiltration of leukocytes. Fungal material was deposited in the terminal airways with a similar pattern in both groups of mice (Figure S1). However, with *A. fumigatus*, some conidia in the lungs appeared swollen or exhibited germ tube formation, while the morphology of *A. versicolor* conidia was unchanged (Figure 3G–I, and Table 1). Conidia from both species were equally able to be cultured from lung homogenates, indicating that viability was not significantly affected (data not shown). These results suggest that the ability of *Aspergillus* conidia to germinate in mouse lung tissue in our model may be species-dependent.

**Table 1:** Summary of Conidia Germination in Lung Tissue.

Exposure Group	# Sections Analyzed	Mean±SEM (/100 conidia)
*A. fumigatus* viable	15	3.933±0.7462
*A. fumigatus* fixed	5	0
*A. fumigatus* heat inactivated	5	0
*A. versicolor* viable	5	0

### Airway CD8^+^, but not CD4^+^, T cell recruitment and IFN-γ-production is decreased in response to non-viable *A. fumigatus* conidia

**Figure 4:**
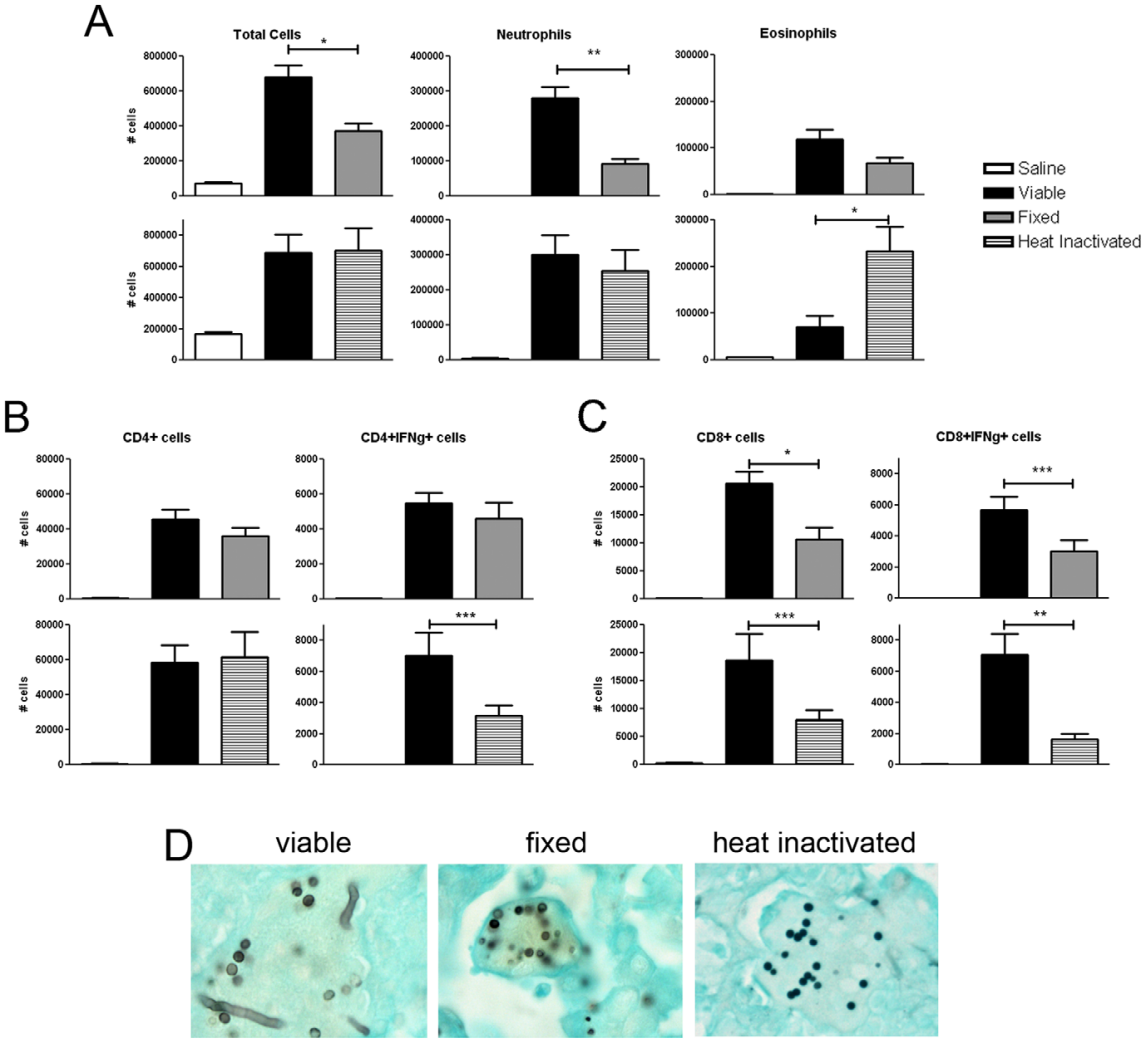
Airway recruitment of IFN-γ-producing CD8^+^ T-cells is decreased in response to *p*-formaldehyde-fixed or heat-inactivated *A. fumigatus* conidia. A, total # of BALF cells, neutrophils, and eosinophils. B, BALF CD4^+^ and CD4^+^IFN-γ^+^ T-cell composition. C, BALF CD8^+^ and CD8^+^IFN-γ^+^ T-cell composition. D, GMS stained (40×) mouse lungs depicting germ tube formation in mice that aspirated viable (left panel), and lack of germ tube formation in mice that aspirated fixed or heat-inactivated conidia (center, right panels). Data depicted in A–C are a summary of two experiments with groups of 5 (saline control) or 10 (viable or non-viable conidia). Histology panels depicted are representative of 5 mice per group with one lung section analyzed per animal. *p<0.01, **p<0.001, ***p<0.05.

Since only *A. fumigatus* demonstrated evidence of lung germination following repeated conidia aspiration, we questioned whether airway immune responses to this species were influenced by metabolic activity of germinating conidia. We therefore examined airway recruitment of T cells in response to inactivated *A. fumigatus*. Paraformaldehyde-fixed or heat-inactivated *A. fumigatus* conidia were aspirated and flow cytometric analysis of BALF cells indicated a decreased total number of cells with fixed, but not heat-inactivated conidia in comparison to viable conidia (Figure 4A, left panels). This decrease was partially attributed to a significant decrease in neutrophils (Figure 4A, top middle panel), while eosinophil recruitment was not significantly affected (Figure 4A, top right panel). However, eosinophils were increased in response to heat-inactivated conidia (Figure 4A, bottom right panel), while neutrophils were unchanged (Figure 4A, bottom middle panel). Interestingly, CD4^+^ T cell recruitment was not decreased in response to conidia inactivated by either method, while CD8^+^ T cells were decreased regardless of method (Figure 4B, left panels, and 4C, left panels, respectively). IFN-γ-producing CD4^+^ T cells were significantly reduced only in response to heat-inactivated conidia (Figure 4B, right panels), while both methods of inactivation resulted in decreased IFN-γ-producing BALF CD8^+^ T cells (Figure 4C, right panels). Little or no BALF T-cell production of IL-4 was detected (data not shown). Histological examination of lung sections indicated that germ tubes or swollen conidia were only visible in mice that aspirated viable conidia, whereas in mice that aspirated non-viable conidia, no signs of lung germination were detected (Figure 4D, E, and Table 1). Together with the data comparing *A. fumigatus* and *A. versicolor* aspiration, these data indicate that airway CD8^+^ T-cell responses to *A. fumigatus* are correlated with germination of conidia in host lung tissue.

### Lung airway memory-phenotype CD8^+^ T-cells are correlated with germination of viable conidia

**Figure 5:**
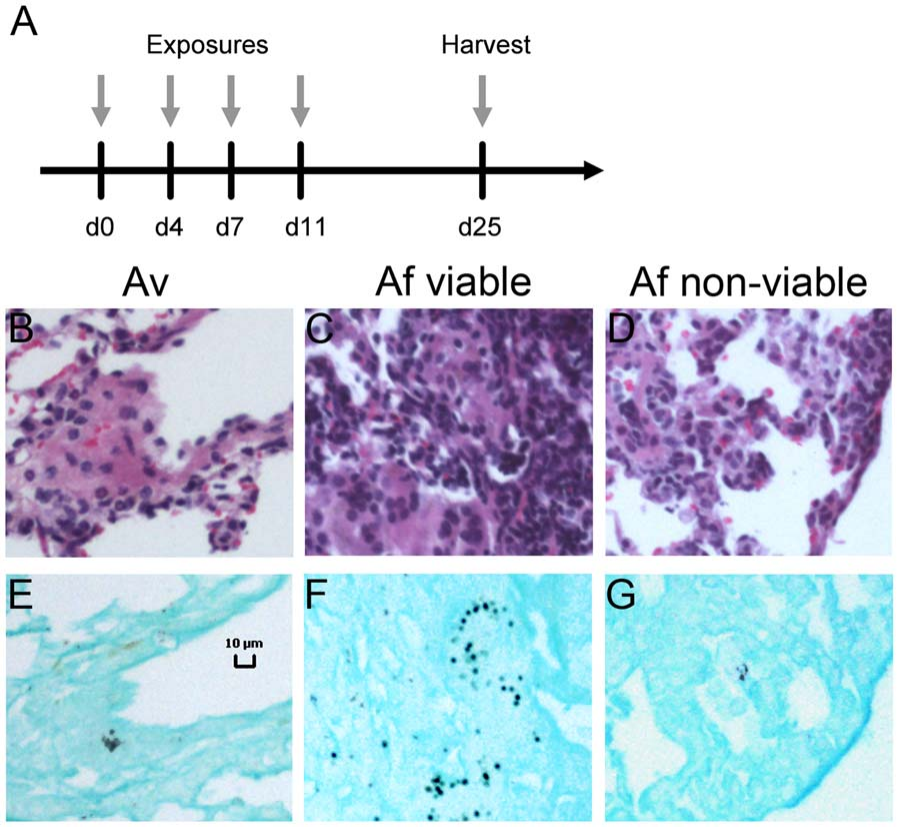
Increased persistence of *A. fumigatus* in lung tissue two weeks after exposure. A, mice aspirated viable or non-viable *A. fumigatus* or *A. versicolor* conidia at the time points indicated, and were sacrificed two weeks after the final aspiration. B–D, H&E staining (20×) of a representative lung tissue sample from each group. E–G, GMS staining (20×) from sections adjacent to H&E stained tissue indicate persistent fungal material. Panels are representative of 5 mice per group with one lung section analyzed per animal.

It is possible that the disparate airway T cell responses we observed were also influenced by differences in the ability of fungal conidia/antigen to persist in the lung tissue after aspiration. Therefore, we performed lung histological examination of tissue from mice that were sacrificed in the absence of a final challenge, two weeks after repeated conidia exposures (Figure 5A). Examination of H&E sections displayed lymphocytic granulomas in lungs of mice that had repeatedly aspirated both viable or non-viable *A. fumigatus* or *A. versicolor* conidia (Figure 5B–E and data not shown). These granulomas frequently contained what appeared to be intact conidia or in some instances fungal debris (Figure 5F–I). However, mice aspirated with viable *A. fumigatus* appeared to display a marked increase in inflammation, granuloma formation, and fungal particles in lung tissue in comparison to non-viable *A. fumigatus* or *A. versicolor* conidia treated mice (Figure 5C,F and data not shown). Therefore, persistence of fungal material is correlated with the ability to germinate in host lung tissue.

**Figure 6:**
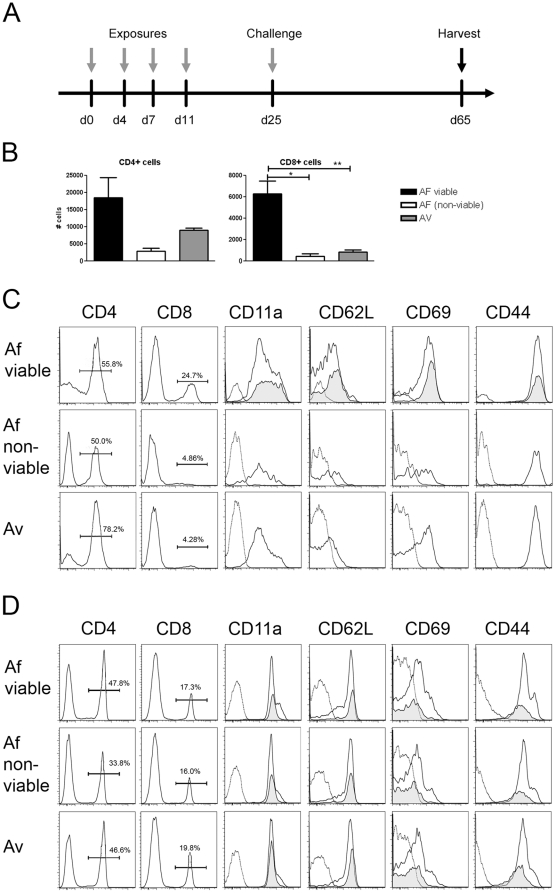
Increased maintenance of airway memory-phenotype CD8^+^ T cells in response to viable *A. fumigatus*. A, mice aspirated viable or non-viable *A. fumigatus* or *A. versicolor* conidia at the time points indicated, and were sacrificed 40 days after the final challenge. B, total # of BALF CD4^+^ and CD8^+^ T cells. Data are a summary of two experiments, 5–10 mice per group. C,D T-cell surface expression of memory markers. Memory surface maker labeled histograms depict both CD4^+^ (open) and CD8^+^ (shaded) T-cell expression where both populations were present. Control (rat-Ig) stained cells are indicated by dotted lines. Panels depicted are representative samples from each group. C, BALF cells. D, MLN cells. *p<0.005, **p<0.001.

In order to examine the persistence of memory airway T cells, we harvested BALF and mediastinal lymph node (MLN) cells from mice at 40 days after the final conidia challenge (Figure 6A). Cell suspensions were then stained for surface markers associated with a lung memory phenotype [21] and analyzed by flow cytometry. Although BALF lung CD4^+^ T cell recruitment appeared different between viable and non-viable *A. fumigatus* or *A. versicolor*-treated mice, the differences were not statistically significant (Figure 6B). However, similar to results at d3 post-challenge, BALF CD8^+^ T cells persisted only in response to viable *A. fumigatus* conidia. Furthermore, BALF T cells expressed surface markers associated with lung T cell memory responses (Figure 6C). BALF T cells were CD11a^hi/lo^CD62L^lo^CD69^hi^CD44^hi^. Both CD4^+^ and CD8^+^ T cells expressed a memory phenotype at d40 after *A. fumigatus* challenge, with only CD4^+^ T cells present in the airways of mice that aspirated *A. versicolor* or non-viable *A. fumigatus*. However, all groups displayed populations of CD4^+^ and CD8^+^ memory-phenotype T cells in the draining lymph nodes (Figure 6D). These data suggest that the germination of *A. fumigatus* conidia at physiological temperature may play a role in the maintenance of airway anti-fungal memory CD8^+^, but not CD4^+^, T-cell responses.

## Discussion

In this study, we examined the airway recruitment of granulocytes and T-cells in response to repeated aspiration of *Aspergillus* conidia and report varied responses to two different species. Airway T-cell responses to the pathogenic fungus, *A. fumigatus*, were characterized by the recruitment of CD4^+^ and CD8^+^ T cells, whereas airway T-cell recruitment to *A. versicolor* was primarily CD4^+^. Furthermore, airway CD4^+^ and CD8^+^ T-cells produced IFN-γ in response to *A. fumigatus*, while fewer airway CD4^+^ T-cells from *A. versicolor*-challenged mice secreted IFN-γ with IL-4-producing cells. Eosinophil recruitment was also significantly increased in the airways of mice that aspirated *A. versicolor*. In our murine model, these results suggest that airway immune responses to repeated aspiration of *A. fumigatus* are skewed primarily towards T_h_1, whereas responses to *A. versicolor* include components of T_h_2 responses. Previous studies have shown that T_h_1 responses are critical for protection from invasive pulmonary aspergillosis, while T_h_2 responses increase susceptibility to *A. fumigatus* infection [14], [22], [23]. Lung eosinophilia has been reported in numerous studies of responses to inhalation and allergic sensitization to *A. fumigatus* extracts and intact conidia [24], [25], [26]. Eosinophilia in response to inhalation of *A. fumigatus* extracts is mediated by T_h_2 cytokines, as airway recruitment is decreased in the presence of antibodies to IL-5 [27]. Recruitment of granulocytes and T-cells to the airways of mice in response to repeated aspiration of *A. versicolor* has not been extensively characterized. It is likely that enhanced lung eosinophilia in response to *A. versicolor* is mediated by increased IL-5. However, we were unable to detect IL-5 in significant amounts in lung homogenates from mice in response to aspiration of conidia (data not shown). In addition to T_h_1 and T_h_2 cytokines, T_h_17 cells have recently been shown to be involved in the pathology of *A. fumigatus*, playing a largely negative role that promotes inflammation and may ultimately impair anti-fungal resistance [28]. Furthermore, In addition to T_h_1 and T_h_2 cells, T_h_17 cells have been shown to be increased in lung tissues in response to repeated intranasal challenge of *A. fumigatus* conidia [20]. Since we have observed important differences in T-cell cytokine production in response to viable and non-viable *Aspergillus* conidia, we believe the role of germination in induction and maintenance of IL-17-producing airway CD4^+^ and CD8^+^ T-cell responses is an important area of future investigation.

Although CD4^+^ T-cells have been shown to confer protection from *A. fumigatus* infection [14], [15], the role of CD8^+^ T-cells has not been examined. IFN-γ-production by CD8^+^ T cells in response to *A. fumigatus* antigens was shown in two studies of blood lymphocytes isolated from healthy human donors [17], [29]. Furthermore, *A. fumigatus* extracts were shown to enhance ovalbumin-specific CD8^+^ T cell responses in T-cell receptor transgenic OT-I mice [30]. Increased airway CD8^+^ T cells have been observed in clinical cases and experimental models of hypersensitivity pneumonitis (HP), and *A. fumigatus* is well-documented as an etiologic agent of HP [31], [32]. Furthermore, lymphocytic granulomas are also prominent in the lung pathology of HP, and were also observed in tissue sections in our study, particularly in response to viable *A. fumigatus*. CD8^+^ T cells confer protection from numerous lung pathogens, including the dimorphic fungus *Cryptococcus neoformans* [33], [34], [35]. Similar to our findings, CD8^+^ T cells have been recently demonstrated to infiltrate murine lung tissue in response to repeated exposures of *A. fumigatus* conidia [20]. Our study advances this observation by more specifically examining recruitment, cytokine production, and the role of germination in induction and maintenance of airway CD8^+^ T-cell responses. However, the role of CD8^+^ T cells in clearance of conidia and protection from *A. fumigatus* infection requires further study, and the specific fungal antigens these cells recognize remain unknown. Airway recruitment of effector CD4^+^ and CD8^+^ T cells and subsequent development and maintenance of airway T-cell memory have been demonstrated in mouse models of pulmonary virus infection [36], [37]. Our results suggest that memory T cells persist in the airways of mice long after exposure to *Aspergillus* conidia, with decreased airway CD8^+^ T cell memory cells in response to *A. versicolor* or non-viable *A. fumigatus*.

Our model suggests that the induction of airway CD8^+^ T cell responses to *A. fumigatus* conidia is mediated in part by the ability to germinate at physiological temperatures. We demonstrated decreased airway recruitment of IFN-γ-producing CD8^+^ T cells in response to non-viable conidia. Furthermore, germ tubes from viable *A. fumigatus* conidia were detected within host lung tissue primarily in macrophages, while no signs of lung germination of *A. versicolor* or non-viable *A. fumigatus* conidia were detected. A previous study by Rivera et al. demonstrated that *A. fumigatus*-specific airway CD4^+^ T-cell production of IFN- γ in response to a single intratracheal challenge of 20×10^8^ heat-inactivated conidia was decreased when compared with viable conidia [19]. In our study, mice subjected to repeated aspiration of 2×10^6^ heat-inactivated, but not paraformaldehyde fixed, conidia displayed a significant decrease in recruitment of IFN-γ-producing CD4^+^ T cells. Our study also used chemically fixed conidia similar to Aimanianda et al. [38], while Rivera et al. exclusively used heat inactivation [19]. Furthermore, our results demonstrate a decrease in airway neutrophil recruitment in response to fixed conidia, and an increase in eosinophils in response to heat-inactivated conidia, suggesting that both methods of inactivation also differ in their stimulation of inflammatory responses. It is possible that heat inactivation and chemical fixation may alter conidial surface antigens differently, and these could affect airway immune responses independently of germination. However, since viable *A. versicolor* conidia also induced less robust CD8^+^ T cell responses when compared with viable *A. fumigatus*, we believe that decreased responses to non-viable conidia are a result of decreased exposure to secreted fungal antigens [8] and inflammatory β-glucans [9] that are induced in germinating conidia.

Although our results suggest that airway CD8^+^ T cell responses in our model may be species-dependent, it is also possible that different fungal isolates may induce distinct responses. In our study, we used *A. fumigatus* Af293, a clinical isolate, and *A. versicolor* ATCC#44408, an environmental isolate. It will be necessary in future studies to examine other clinical and environmental isolates to determine if these differences are indeed species-dependent, or if the variation observed is based on the ability of each isolate to invade or colonize host tissue.

These data suggest a correlation between the ability of conidia to germinate *in vivo* and the induction of airway immune responses to *Aspergillus* aspiration. Although *A. fumigatus* is not considered an intracellular pathogen, the ability of conidia to germinate within lung macrophages may be sufficient for induction of CD8^+^ T-cell responses that are characteristic of responses to intracellular infection. The results of this study add to the growing body of evidence suggesting that the adaptive immune system possesses the ability to discriminate between microbes and other environmental antigens by determining their invasive potential, and subsequently programming appropriate responses to maximize protection from infection while minimizing damage to host tissue.

## Materials and Methods

### Growth and handling of fungi

Fungal isolates (*A. fumigatus*, strain Af293, *A. versicolor*, strain 44408) were purchased from the Fungal Genetics Stock Center (FGSC) and the American Type Culture Collection (ATCC), respectively. Fungi were grown on malt extract agar (MEA) plates at 25°C. Fungal spores were isolated from cultures kept in the dark at room temperature (RT) for 14 days, by applying 1 g of glass beads (0.5 mm, Braun-Melsungen, Melsungen, Germany) and gently shaken. The bead/conidia mixture was collected into a tube and suspended in 1 mL sterile phosphate buffered saline (PBS). The beads were vortexed and the supernatant containing the conidia was removed and counted with a hemacytometer. Conidia were subsequently resuspended to a concentration of 4×10^7^ conidia/ml. For inactivation, *A. fumigatus* conidia were fixed according to the method of Aimanianda et al. [38]. Briefly, 1 mL of a 4% paraformaldehyde solution was added to the bead/conidia mixture, vortexed, and incubated overnight at 4°C. Fixed conidia were centrifuged, then washed in 0.1 M ammonium chloride, followed by another wash and resuspension in 1 mL sterile PBS. As an alternative method of inactivation, conidia were autoclaved for 30 minutes [19]. Conidial inactivation by either method resulted in >99.9% reduction in viability by serial dilution. Vortexing glass beads with conidia in solution did not significantly alter viability.

### Mouse aspiration, sacrifice, histological staining, and collection of BALF

Female BALB/c mice, aged 5–7 weeks, were obtained from Jackson Laboratory (Jackson Laboratory, Bar Harbor, ME) and allowed to rest approximately one week before initial exposures. The NIOSH animal facility is an environmentally controlled barrier facility fully accredited by the Association for the Assessment and Accreditations of Laboratory Animal Care International. Fungal suspensions were delivered by involuntary aspiration as previously described [39]. Briefly, mice were anesthetized with isoflurane and suspended on a slant board. The tongue of the animal was held in full extension as a 50 µL suspension of 2×10^6^ spores in PBS was placed at the base of the tongue. The tongue was restrained briefly for approximately two breaths while the mice inhaled the conidial suspension, after which anesthetized mice were returned to the cage and allowed to recover. To examine lung deposition of aspirated conidia, mice were sacrificed one hour after aspiration, and lungs were inflated with air, and tissue was fixed for histological analysis. To assess responses to repeated exposures, mice aspirated conidia twice a week for two weeks (Figure 1A, Figure 5A, and Figure 6a). Some mice were challenged after a two week rest and others were harvested with no final challenge. After the final challenge (d3 or d40; Figure 1A, Figure 5A, and Figure 6a) mice were sacrificed with an intraperitoneal injection of sodium pentobarbital and the lungs were perfused with 10 mL PBS prior to collection of bronchoalveolar lavage fluid (BALF). For histological preparation, lungs were perfused with 5 mL of PBS followed by further perfusion and inflation of the lungs with 10% buffered formalin phosphate (5 mL and 1 mL, respectively) (Fisher Scientific, Fair Lawn, NJ). Tissue processing, embedding, and hematoxylin and eosin (H&E) staining was performed by the West Virginia University Tissue Bank (Morgantown, WV). To detect fungal germination in the lungs, Grocott's Methanamine Silver (GMS) stain was performed on lung sections by the tissue pathology laboratory of the Pathology and Physiology Research Branch (CDC-NIOSH, Morgantown, WV). To obtain the frequency of germinated conidia in lung tissues, 100 conidia from one section per sample were randomly counted for signs of germination. Germinating conidia were defined by conidial swelling (2–3× normal size) or by the formation of germ tubes. BALF was collected by exposing and nicking the trachea followed by insertion of a catheter tied off with suture to prevent leakage. A syringe containing 1 mL of PBS was attached to the tracheal catheter, with the liquid injected into the lungs and subsequently removed. This process was repeated until 3 mL of BALF was collected. All animal procedures were approved by the National Institute of Occupational Safety and Health Animal Care and Use Committee (protocol# 08-ST-M-015).

### Flow cytometric analysis of bronchoalveolar lavage fluid

All reagents were obtained by BD biosciences (BD biosciences San Jose, CA) unless otherwise specified. BALF cell composition was determined by flow cytometric analysis of recovered lavage cells in suspension. BALF was centrifuged for 5 min at 1500 rpm, the supernatant removed, and the cell pellet resuspended and washed in 1 mL of FACS buffer (PBS, 5% fetal bovine serum, 0.05% sodium azide). The washed pellet was resuspended and stained in FACS buffer, 10% rat serum, Fc-receptor blocking antibody (clone 24G2) and the following antibodies: rat anti-mouse Ly-6G FITC, rat anti-mouse Siglec-F PE, pan-leukocyte rat anti-mouse CD45 PerCP, and rat anti-mouse CD11c APC. After staining for 30 minutes in the dark on ice, cells were washed and fixed with BD Cytofix, and resuspended in FACS buffer. Populations of cells were evaluated by flow cytometric analysis on a BD FACSCalibur, or in the case of memory T cells, on a BD LSRII (BD Biosciences, San Jose, CA). Neutrophils were defined as CD45^hi^Ly-6G^hi^CD11c^lo^, eosinophils were defined as Ly-6G^lo^Siglec-F^hi^CD11c^lo^ and alveolar macrophages were Ly-6G^lo^Siglec-F^hi^CD11c^hi^, as previously reported [40]. In a separate tube, BALF T cells were quantified using rat anti-mouse CD4 FITC and CD4 PerCp antibodies. Total numbers of each cell population were obtained by multiplying the frequency of the specific population by the total number of BALF cells recovered for each animal. For examination of airway memory cells, BALF cells were harvested from mice 40 days after the final aspiration and stained for airway memory T cell markers [21]; these included rat anti-mouse CD4 APC-Cy7, CD8 FITC, CD11a PE, CD62L PerCp Cy5.5, CD69 PE-Cy7, and CD44 APC.

### Intracellular cytokine staining

All reagents were obtained by BD biosciences unless otherwise specified. T-cell cytokine production was determined by fluorescent intracellular cytokine staining (ICS) as previously described [41]. Briefly, the BALF suspension was centrifuged for 5 min at 1500 rpm and washed in 1 mL of complete medium. The supernatant was discarded and a solution of Leukocyte Activation Cocktail with GolgiPlug in 0.2 mL complete medium was added to each sample for stimulation of cytokine production and simultaneous inhibition of cytokine secretion. Cells were incubated at 37°C for 4 hrs. After incubation, the cells were washed in FACS buffer and stained for flow cytometry using rat-anti-mouse CD4 PerCP and rat-anti-mouse CD8 FITC on ice. After a 30 min incubation, cells were washed in FACS buffer and centrifuged, and cell pellets were resuspended in BD Cytofix/Cytoperm for 15 minutes to allow for fixation and permeabilization required for subsequent intracellular cytokine staining. Cells were washed with 1 mL BD Permwash, and resuspended in Permwash. Each sample was equally divided into two tubes and stained with rat-anti-mouse IFN-γ APC and rat-anti-mouse IL-4 PE, or with control isotype antibodies (eBioscience, San Diego, CA). Cell populations were analyzed on a BD FACSCalibur, with lymphocytes gated on the basis of low forward and side scatter, then subsequently gated on CD4^+^ or CD8^+^ populations to determine intracellular expression of cytokines.

### Data analysis methods

Analysis of flow cytometric samples was performed with FlowJo software (TreeStar, Ashland, OR). GraphPad Prism was used for generation of graphs and figures and for statistical analyses (GraphPad Software, La Jolla, CA). Unpaired t-tests were performed to measure statistical significance, and differences between experimental groups that resulted in a p value of less than 0.05 were considered significant.

## Supporting Information

Figure S1 **Lung deposition of fungal conidia.** Mice were sacrificed one hour after a single aspiration with 2×10^6^ conidia from *A. versicolor* or *A. fumigatus*, and lung tissues were inflated with air and fixed with formalin. Paraffin-embedded sections were GMS stained to determine the pattern of lung deposition. A, saline control. B, *A. versicolor*. C, *A. fumigatus*. Panels are representative of 3 mice per group with one lung section analyzed per animal.
(TIF)
