# Supplementary figures and images for: Role of Germination in Murine Airway CD8+ T-Cell Responses to Aspergillus Conidia
Source: PLoS One. 2011 Apr 13;6(4):e18777. doi: 10.1371/journal.pone.0018777 (PMC3076443; doi:10.1371/journal.pone.0018777)

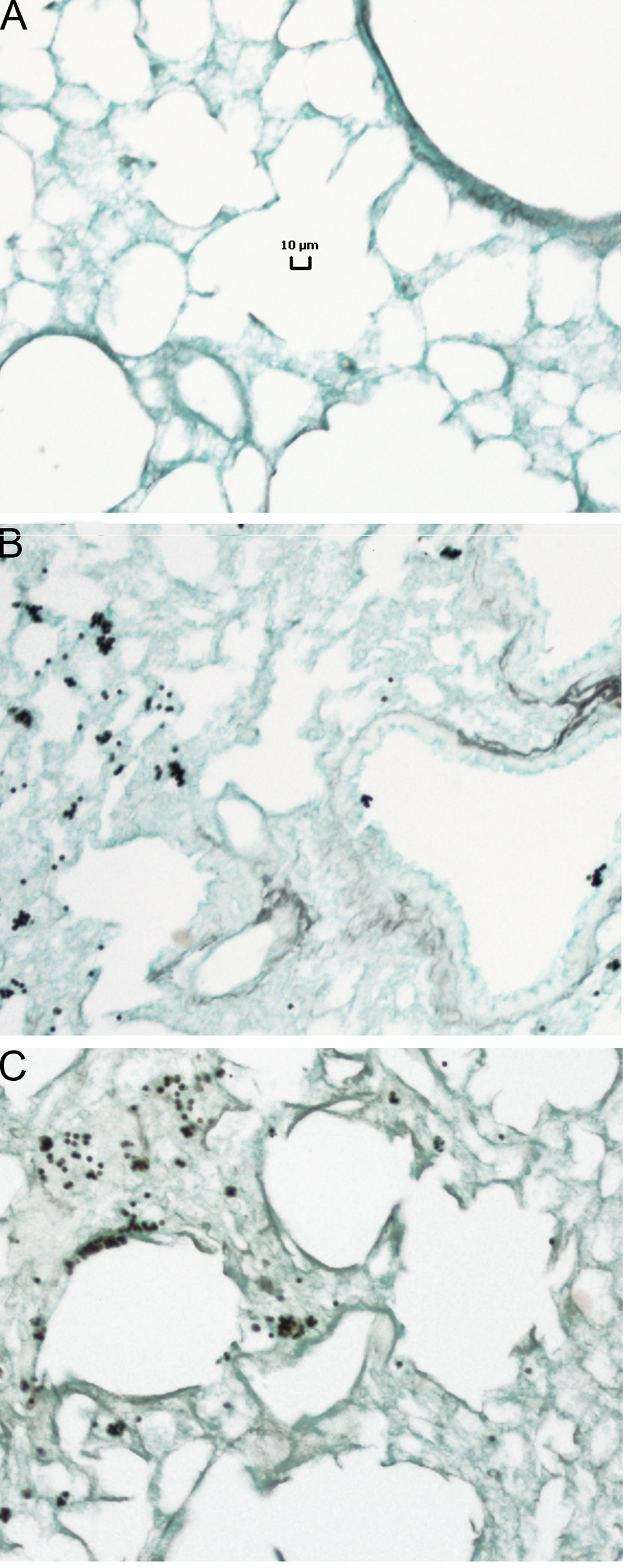

Supplement: Figure S1 — Lung deposition of fungal conidia. Mice were sacrificed one hour after a single aspiration with 2×10^6^ conidia from A. versicolor or A. fumigatus, and lung tissues were inflated with air and fixed with formalin. Paraffin-embedded sections were GMS stained to determine the pattern of lung deposition. A, saline control. B, A. versicolor. C, A. fumigatus. Panels are representative of 3 mice per group with one lung section analyzed per animal. (TIF) [file pone.0018777.s001.tif]
